# Goblet cell breakdown: transcriptomics reveals *Acinetobacter baumannii* early and robust inflammatory response in differentiated human bronchial epithelial cells

**DOI:** 10.1186/s12929-025-01159-1

**Published:** 2025-07-09

**Authors:** Daniela Scribano, Claudia Tito, Astri D. Tagueha, Martina Pasqua, Luciana De Angelis, Francesco Fazi, Dolores Limongi, Marta De Angelis, Lucia Nencioni, Anna Teresa Palamara, Cecilia Ambrosi

**Affiliations:** 1 Department of Public Health and Infectious Diseases, Sapienza University of Rome, 00185 Rome, Italy; 2 Department of Anatomical, Histological, Forensic & Orthopedic Sciences, Section of Histology & Medical Embryology, Sapienza University of Rome, 00161 Rome, Italy; 3 Department of Biology and Biotechnologies “Charles Darwin”, Institute Pasteur Italia, Sapienza University of Rome, 00185 Rome, Italy; 4 Department of Promotion of Human Sciences and Quality of Life, San Raffaele Open University, 00166 Rome, Italy; 5 Laboratory of Microbiology, IRCCS San Raffaele Roma, 00143 Rome, Italy; 6 Department of Public Health and Infectious Diseases, Laboratory Affiliated to Institute Pasteur Italia-Cenci Bolognetti Foundation, Sapienza University of Rome, 00185 Rome, Italy; 7 Laboratory of Virology, Department of Molecular Medicine, Sapienza University, Rome, Italy; 8 Department of Infectious Diseases, Istituto Superiore Di Sanità, 00185 Rome, Italy

**Keywords:** Air liquid interface epithelium, 2D co-culture, Infection model, *Acinetobacter baumannii*, Host–pathogen interaction, Transcriptome

## Abstract

**Background:**

The airway epithelium represents the first line of defense of the lungs, functioning both as a physical barrier as well as an active immune modulator. However, in the last years, pneumonia caused by the opportunistic pathogen *Acinetobacter baumannii* have become difficult to treat due to the increase of the number of extensively drug resistant strains. In this study, we report for the first time the use of an *ex vivo* air–liquid interface (ALI) model of differentiated human bronchial epithelial cells to unravel the early response to *A. baumannii* infection.

**Methods:**

Epithelial integrity, tissue architecture, and goblet cell function were assessed through FITC-dextran permeability assays, hematoxylin and eosin staining, and indirect immunofluorescence. Transcriptomic profiling was performed to characterize host gene expression changes.

**Results:**

Initial tissue damage began as early as at 4 h post-infection (hpi); at 24 hpi, goblet cell hypertrophy, reduced mucin secretion, and compromised epithelial integrity were highly evident. Transcriptomic data at 4 hpi revealed 668 differentially expressed genes (441 upregulated, 227 downregulated), mainly involved in a strong pro-inflammatory response and characterized by IL-8/CCL20-driven neutrophil recruitment and type 2 cytokine activation (IL-4, IL-13). Noteworthy, genes related to cytoskeletal organization, adhesion, and extracellular matrix remodeling were significantly altered, suggesting a bacterial mechanism to enhanced tissue dissemination. The PI3K-Akt survival pathway was inhibited, with downregulation of *PIK3R1* and *PIK3R2* genes, implying the induction of apoptosis/cell death and epithelial damage. Our findings are in agreement with previous *in vivo* studies, further strengthening the value of our ALI model in mimicking the early infection response of bronchial cells to *A. baumannii* infection.

**Conclusion:**

Our data highlight the early molecular mechanisms underlying *A. baumannii* pathogenesis and open new avenues for future investigations for therapeutic interventions.

**Graphical Abstract:**

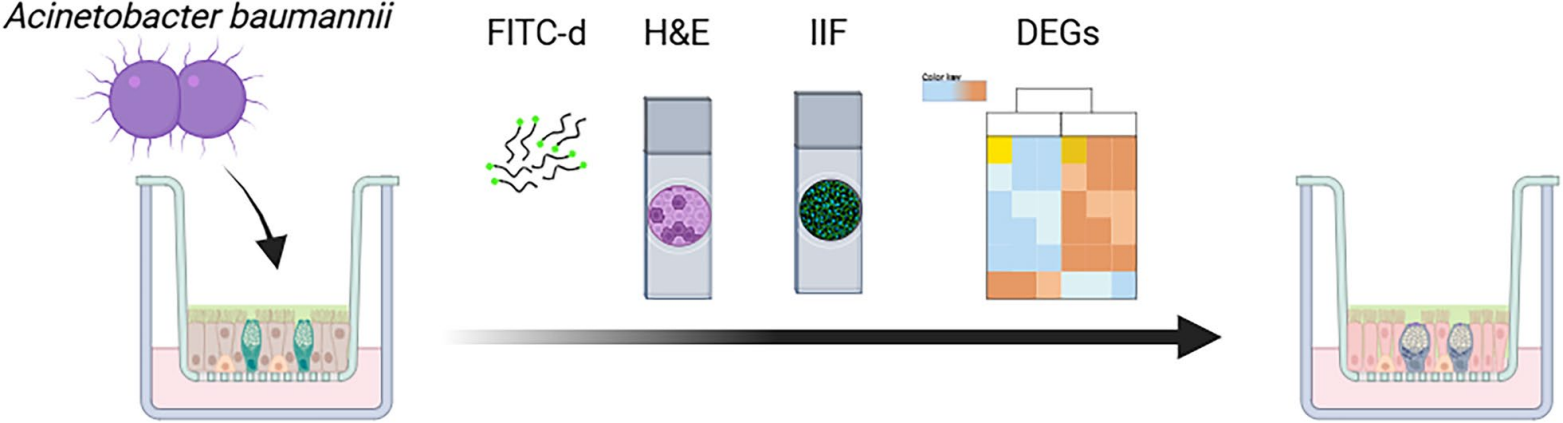

**Supplementary Information:**

The online version contains supplementary material available at 10.1186/s12929-025-01159-1.

## Introduction

*Acinetobacter baumannii* is an opportunistic pathogen that causes severe infections in immunocompromised individuals, mainly pneumonia [1, 2]. If untreated, *A. baumannii* infections can lead to high rates of morbidity and mortality [3]. However, with the increase of the number of extensively drug resistant strains the therapeutic options to eradicate this pathogen are dramatically reduced [1–3]. The World Health Organization reports that carbapenem-resistant *A. baumannii* strains remain critical microorganisms urgently needing new therapeutic options (https://www.who.int/publications/i/item/9789240093461).

Adhesion is the first and most critical step for infection; *A. baumannii* strains have a plethora of appendages and adhesins allowing bacterial attachment to abiotic and biotic surfaces [4, 5]. Key adhesion factors for the interaction with host epithelial cells include chaperone-usher pathway pili (Csu pili), the *Acinetobacter* trimeric autotransporter (Ata), the biofilm-associated protein (Bap), the filamentous hemagglutinin C (FhaC), the outer-membrane protein A (OmpA), the TonB-dependent outer membrane receptor (BauA), and the ferrous iron transport protein FeoA, just to mention the most studied [2, 6]. Transcriptome studies in *ex vivo* and *in vivo* models reveal upregulation of genes linked to antibiotic resistance, iron acquisition, and surface-associated properties, among different isolates [7, 8]. Several modifications of *A. baumannii* transcriptome occur to improve its fitness and survival during infection within the host [9, 10]. It also undergoes mucoid conversion over time during pulmonary infections to further enhance survival in this context by avoiding phagocytosis and pulmonary clearance [11]. However, few studies have analyzed the host response to colonization of *A. baumannii* [12]. One *in vivo* study shows that *A. baumannii* establishes an acute lethal pneumonia after 24 h, supported by high bacterial burdens, severe inflammatory cells infiltration and lung damage [13] Understanding the interaction between *A. baumannii* and epithelia could reveal new diagnostic biomarkers and strategies to combat pneumonia caused by *A. baumannii*. Advanced *in vitro* infection models, such as air–liquid interface (ALI) culture models, replicate the human lung environment and can be exploited to dissect the different stages of microbial infections [14–17]. Indeed, these cells form a pseudostratified epithelium, including basal, ciliated, and mucus-secreting goblet cells, closely mimicking the native human airway epithelium [14–17]. Derived from 2D airway organoids or primary cells, these cultures have been used to study *Pseudomonas aeruginosa* infections and replicate important aspects of *in vivo* infection [18, 19]. Therefore, the present study aimed to establish how human respiratory epithelia respond to *A. baumannii* colonization using advanced *in vitro* models that mimic host physiological conditions. By using for the first time differentiated primary human airway epithelial cells (2D ALI) derived from a healthy donor, we established how human bronchial cells respond to *A. baumannii* initial colonization steps by performing histological, immunofluorescence, and transcriptome sequencing (RNA-seq) analyses. Herein, we demonstrate that *A. baumannii* rapidly induces a robust pro-inflammatory transcriptomic response driven by chemokine signaling, coupled with early disruption of epithelial barrier integrity, goblet cell dysfunction, and increased permeability.

## Materials and methods

### Bacterial growth conditions and cell cultures

The *A. baumannii* AB5075-UW strain used in this study was provided by BEI Resources (Manassas, VA). Routine growth and plating were carried out in Luria–Bertani broth (LB) and 1.5% agar plates (Difco, Milan, Italy). A single opaque colony was inoculated in LB and grown at 37 °C with vigorous shaking (200 rpm) to the mid-exponential phase (optical density (OD) at 600 nm ≈ 0.8.

Normal human bronchial epithelial cells (NHBE) (CC-2540, Lonza, Italy, ID 45627) were seeded at a density of 1.7 × 10^5^ cells in a T25 flask, following manufacturer’s instructions. Expansion phase was achieved using PneumaCult^™^ ExPlus medium (STEMCELL Technologies, Italy). Once 50% confluent, cells were washed, and seeded onto 0.4 µm pore, PET 24 well Transwell^®^ inserts (Greiner Bio-one, Italy) at a final density of 3.3 × 10^4^/Transwell. After reaching confluency, the apical media were aspirated (air-lifted), and cells were allowed to differentiate in air liquid interface for 4 weeks in PneumaCult^™^ ALI differentiation media supplemented with 0.2% heparin, 5% hydrocortisone, and 0.2% Pen/strep and amphotericin B (all STEMCELL Technologies, Italy). The day before infection, Transwell inserts were washed twice, and the growth medium, depleted of antibiotics and antimycotics, was replaced in the basal chamber.

### Infection of 2D ALI

2D ALI were washed twice with Dulbecco phosphate-buffered saline (PBS) to remove the produced mucus. The bacterial culture was washed twice in DMEM-F12, diluted, and 20 μl were used to infect the apical side of bronchial epithelia to reach a multiplicity of infection (MOI), as indicated. To estimate the number of cells on inserts, we referred to a previous published study, which adopted identical experimental conditions [20]. Infected and not infected 2D ALI were centrifuged at 200 × *g* for 10 min, and incubated at 37 °C with 5% CO_2_ for 4 and 24 h. Four sets of infected and uninfected 2D ALI were assayed. Parallel uninfected cells exposed to 20 µl of DMEM-F12 served as negative controls.

### Bacterial adherence

After 4 and 24 h of incubation, a set of 2D ALI were washed twice with PBS to remove unadherent bacteria; then, 200 μl of cold distilled water were added, incubated 5 min at room temperature (RT), and the transwell surface was scraped to recover bacteria. Serially diluted lysates were plated on LB agar plates to determine the colony forming units (CFU)/ml.

### Hematoxylin–eosin (H&E) and periodic acid–Schiff (PAS) staining

The apical and basal chambers of a second set of 2D ALI was washed with PBS, and fixed with 10% buffered formalin overnight at 4 °C. Paraffin-embedding of ALI tissue, were sectioned (10 µm), and stained either with H&E or PAS, following a published protocol [21].

### Immunofluorescence

A third set of 2D ALI was washed with PBS, and fixed with paraformadehyde 4% for 20 min at RT. After the washing steps, 2D ALI were permebilized with 0.5% Triton-X 100 for 25 min, blocked with 3% normal goat serum-1% bovine serum albumin (BSA), and incubated overnight with 1: 500 rabbit polyclonal anti-MUC5AC (Abcam AB198294), and 1:250 mouse monoclonal anti-Acetyl-tubulin antibodies (Santa Cruz SC-23950). In parallel, 2D ALI were stained with 1:100 rabbit polyclonal anti ZO-1 (Invitrogen PA5-28869), and 1:250 mouse monoclonal anti-*A. baumannii* antibodies (gift of Prof. B.B. Singer). Secondary anti-rabbit Alexa Fluor 488 (green) and anti-mouse Alexa Fluor 546 (red) antibodies were used 1:250, following manufacturer’s instructions (Invitrogen, Italy). Finally, the nuclei were stained with Hoechst 33342 (Life Technologies) for 10 min in PBS, washed and mounted in VECTASHIELD antifade mounting media (#H1000, Vector Laboratories). Images were acquired using a Zeiss LSM 900 confocal laser scanning microscope using ZEN software (Zeiss).

### RNA isolation, RNA-seq and quantitative reverse-transcription PCR (qRT-PCR)

A fourth set of 2D ALI was used for RNA extraction. RNA was isolated at 4 h post-infection, using TRIzol reagent (Invitrogen, Italy) and Direct-zol RNA kit (Zymo Research, CA, USA), following manufacturer instructions. DNase I (Zymo Research, CA, USA) was used to remove genomic DNA. The quality and quantity of extracted RNA was evaluated by measuring the absorbance ratio A_260_/A_280_ and 1.5% agarose gel electrophoresis, respectively. RNA integrity was measured using the Qubit 4.0 Fluorometer with the RNA IQ Assay kit following the corresponding manufacturers’ standard protocols (Thermo Fisher Scientific, Italy). Frozen samples were sent for library preparation and RNA sequencing to BMR Genomics sequencing service (BMR Genomics srl, Padova, Italy). RNA integrity for library preparation was determined by analysis of extracted total RNA using a 2100 Bioanalyzer (Agilent Technologies) with RNA 6000 NanoChip. RNA concentrations were measured using Qubit RNA Assay Kit. Libraries were prepared from total RNA according to manufacturer instructions with Illumina Stranded mRNA Prep kit. Libraries quality were evaluated by size analysis on 2100 Bioanlyzer (Chip DNA HS) and concentrations were determined using Qubit DNA HS assay kit (Thermo Fisher). Sequencing was performed on Illumina Novaseq 6000 in the 150PE format. Expression of selected genes was determined by reverse transcriptase polymerase chain reaction (RT-PCR) to validate RNA-Seq results. RNA (1 μg) was converted to cDNA with the High Capacity cDNA Reverse Transcription Kit (Applied Biosystems), following manufacturer’s instructions. The RT-PCR analysis was performed using 2 μl of cDNA samples as template with primers reported in Table S1. The expression of each target gene was normalized to *GAPDH* rRNA by using the 2^−ΔΔCt^ method [22]. The assay was conducted in triplicate; means and standard deviations were calculated for each group.

### ALI permeability assay

The integrity of tight junctions of the pseudostratified cell culture was determined chemically by the use of 4 kDa fluorescein isothiocyanate (FITC)-dextran, corresponding to a molecular radius of 14 Angstrom (Sigma-Aldrich, Italy). Briefly, 200 μl of a solution containing 2 mg/ml of FITC-dextran in HBSS was added into the apical chamber, while 500 μl of HBSS was added to the basal chamber. ALI cultures were incubated at 37 °C and 5% CO_2_ for 2 h. Samples from the basolateral medium for each insert were analyzed in triplicate by fluorescence measurement at 4 and 24 hpi, with excitation at 492 nm and emission at 520 nm, using a CLARIOStar microplate reader (BMG Labtech, Offenburg, Germany) in 96-well black microtiter. The Adjust Gain setting was calibrated using a 2 mg/ml solution to standardize all sample readings. Medium without FITC-dextran was used as a blank, while three empty Transwells served as controls for 100% permeability.

### Bioinformatic analyses

Reads preprocessing was performed by using fastp v0.20.0 [23], applying specific parameters to keep only high quality data: qualified_quality_phred = 20, unqualified_percent_limit = 30, average_qual = 25, low_complexity_filter = True, complexity_threshold = 30. Mean value of uniquely mapped reads were 89%, unmapped reads 6% per sample. Passing filter reads were mapped to the genome reference (*Homo sapiens*) using STAR v2.7.9.a [24] with standard parameters, except for sjdbOverhang option set on read length. Genome and transcripts annotation provided as input were downloaded from v105 of Ensembl repository. Alignments were then elaborated by RSEM v1.3.3 [25], to estimate transcript and gene abundances. Subsequently, the sample-specific gene-level abundances were merged into a single raw expression matrix applying a dedicated RSEM command (rsem-generate-data-matrix). Genes with at least 10 counts in N samples were then selected, where N corresponds to the sample number in the smallest experimental group. Differential expression was computed by edgeR [26] from raw counts in each comparison. Multiple testing controlling procedure was applied and genes with a FDR ≤ 0.05 and logFC >|0.5| were considered differentially expressed. Annotation of differential expressed genes was performed using the bioMart package [27] into R 4.3, querying available Ensembl Gene IDs and retrieving Gene Names and Entrez gene IDs. In order to create the heatmap, the R library *(pheatmap)* with an unbiased hierarchical system was used. Obtained differentially expressed genes (DEGs) were used for GO analyses through the *clusterProfiler* library [28], and categorized into biological process (BP), molecular function (MF), and cellular component (CC). The same genes were used for pathway enrichment analyses in the Reactome database using the *ReactomePA* library [29]*.* Metapathway was generated using genes selected from enrichment pathways describing inflammation, apoptosis, and cell–cell interaction, and processed in STRING to extract experimentally validated and database interactions. Cytoscape [30] was used to visualize the corresponding network graphs for each process.

### Video microscopy

A Video of the differentiated epithelia was recordered under a light microscope (Motik AE21 microscopy, Italy) at 10 × magnification at 30 frames per second with a resolution of up to 1920 × 1080 pixels (Samsung Galaxy S20 FE).

### Statistical analysis

A total of four independent experiments were performed (n = 4). Normal distribution was determined with the Shapiro–Wilk test. Statistical significance was determined using the unpaired Student’s t test using Prism software v. 8.0.2 (GraphPad, USA). *P* values < 0.05 were taken as being statistically significant. The comparison of gene expression data by RT-PCR and RNA-seq showed a significant Pearson correlation coefficient between the results of the two approaches (*P* < 0,0001, R squared = 0,9771).

### Shi ***Data availability***

The RNA-seq data of this study have been deposited in NCBI underBioProject No. PRJNA1219108.

## Results

### Establishment of air–liquid interface (ALI) cultures to study *A. baumannii* Pathogenesis Mechanisms

**Fig. 1:**
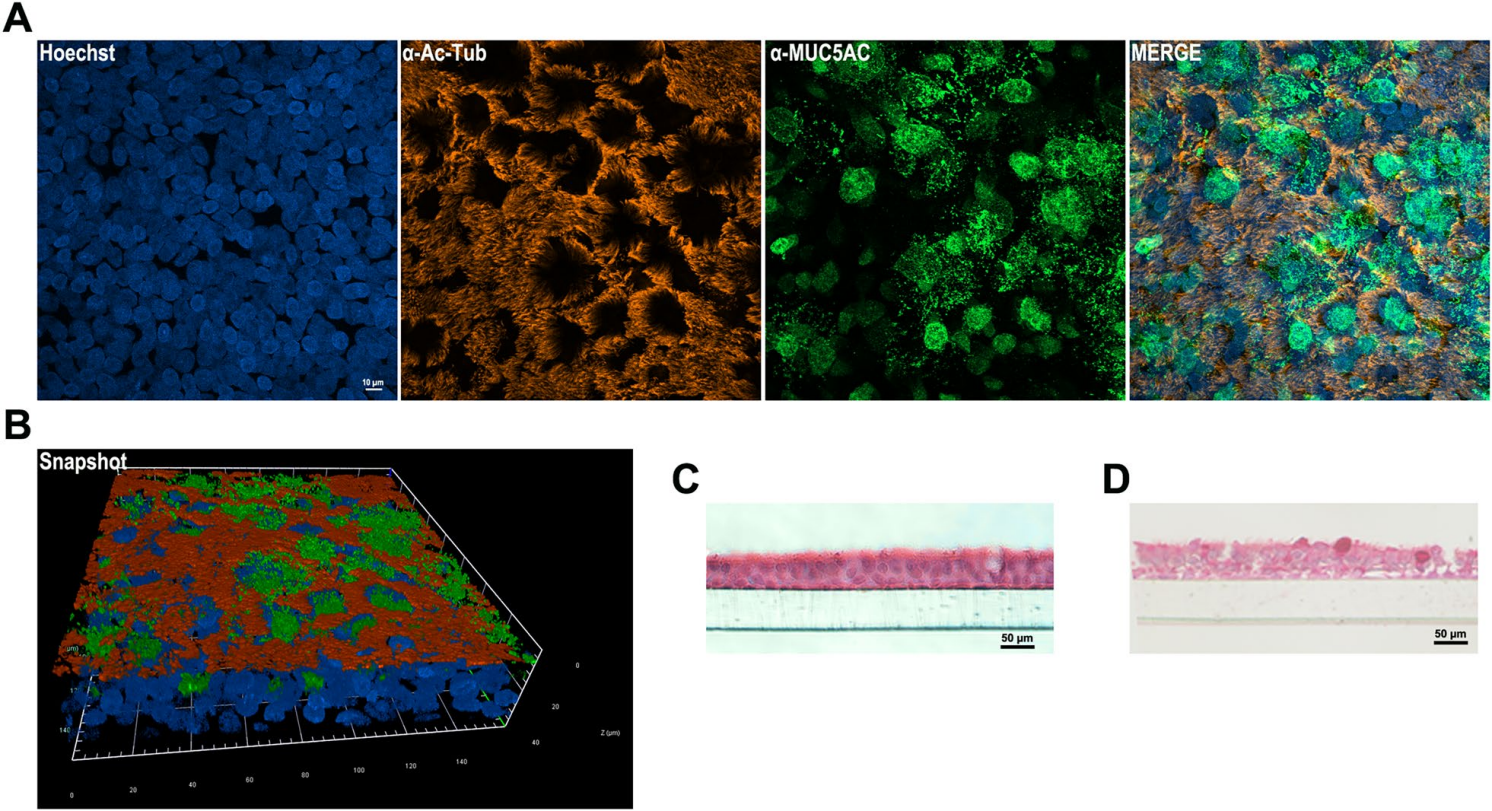
Characterization of differentiated NHBE cells cultured at the air–liquid interface (ALI). 2D ALI cultures were grown for 28 days, before being analyzed. **A** After 28 days of air exposure, the pseudostratified epithelium was stained with specific antibodies to visualize ciliated cells with well-developed apical projections (acetylated tubulin, red), and sparse goblet cells secreting mucin-5 subtype AC- (MUC5AC, green). Nuclei were counterstained with Hoechst 33342 (blue). **B** An image illustrating the spatial organization and differentiation of ciliated and goblet cells within the epithelium. **C**, **D** Representative histological image of tangential sections of the epithelium stained with H&E and PAS, respectively. Three independent experiments using cells from the same donor were performed. Scale bar sizes are indicated in the images

We aimed to investigate the interaction between *A. baumannii* and the airway epithelium during early and late infection. To achieve this, we established ALI cultures using Normal Human Bronchial Epithelial (NHBE) cells derived from a healthy human donor. The cells were cultured on Transwell inserts and allowed to differentiate for 28 days, as detailed in the Materials and Methods section. Immunofluorescence staining of NHBE cells on Transwell inserts confirmed the expression of key markers: acetylated tubulin for ciliated cells and mucin-5 subtype AC (MUC5AC) for goblet cells (Fig. 1A and B). H&E staining of tangential sections of ALI culture highlighted the presence of apical cells mostly columnar in shape, featured with multiple cilia (Fig. 1C), while PAS staining detailed a physiological distribution of mucus-secreting goblet cells (Fig. 1D). Video 1 shows the fully functional pseudostratified tissues, with highly active cilia on their surface, which coordinately beat and move secreted mucus layer. Tightness of the pseudostratified epithelium was tested by measuring the passage of 4 kDa FITC-dextran. The flux of dextran from the upper into the lower compartment, was significantly reduced by the four week-ALI-matured epithelial layer in comparison to the cell-free synthetic Transwell membrane. These findings confirm the successful differentiation of a functional pseudostratified airway epithelium that closely resembles the native human bronchial epithelium, making it well-suited for studying host–pathogen interactions.

### *A. baumannii* induces swelling in goblet cells

**Fig. 2:**
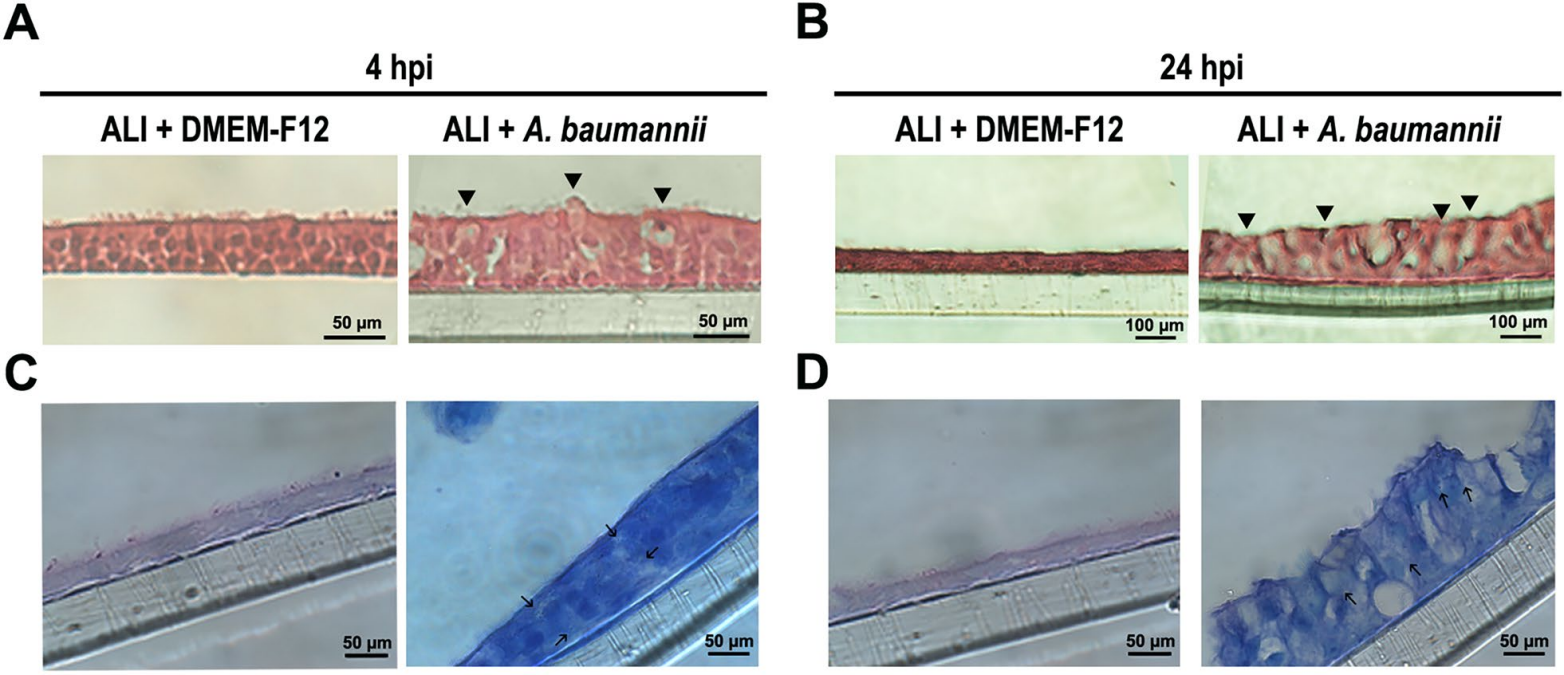
Histology of ALI infected with *A. baumannii*. A total of 20 µl of bacteria resuspended in DMEM-F12 was used for infection. Non-infected ALI cultures were treated with an equivalent volume of DMEM-F12 as a negative control. **A**, **B** Representative images of infected and unifected ALI cultures stained with H&E at 4 h post-infection (hpi) and 24 hpi, respectively, as indicated. **C**, **D** Representative images of the same sections shown in panels (**A** and **B)**, stained with Giemsa. Three independent experiments using cells from the same donor were performed. Scale bar sizes are indicated in the images

The differentiated ALI culture was employed to examine the temporal progression of colonization and the cellular responses of the epithelium during early infection. Thus, 2D bronchial epithelium was apically infected with *A. baumannii* AB5075 at a multiplicity of infection (MOI) of 100. A preliminary time-course infection experiment was performed to evaluate histological changes in the ALI culture following *A. baumannii* infection. Based on these observations, 4 hpi was selected for transcriptomic analysis as early epithelial alterations were detectable at this stage, while 24 hpi was selected to investigate the progression of infection and tissue damage over time. At 4 and 24 h post-infection, the adherent bacteria were assessed by measuring the number of colony forming units (CFU)/ml recovered from the ALI culture. A 2-log reduction in the bacterial count compared to the initial inoculum was recorded, which remained stable over time. Thus, no significant replication of *A. baumannii* was observed at either time point. In accordance with the membrane size (0.4 µm), no bacteria could be recovered from the basal chamber. The histological staining of tangential ALI sections with H&E at 4 hpi revealed that goblet cells appeared lightly swollen, grainy, and irregular in infected cultures compared to non-infected counterparts (Fig. 2A). These changes became more evident and intense at 24 hpi, exibiting a swollen and hypertrophic phenotype (Fig. 2B). The same effect was shown with ALI culture infected with MOI 2 and 5 (Supplementary material Fig. S1). Moreover, Giemsa staining showed that during the first 4 h, the majority of bacteria were closer to goblet cells than to ciliated cells (Fig. 2C). By 24 h, most bacteria remained near goblet cells, although some were observed near ciliated cells, potentially due to the extent of damage to the pseudostratified epithelium (Fig. 2D). Thus, *A. baumannii* caused severe disruption of goblet cells, impacting the overall architecture of ALI cultures.

**Fig. 3:**
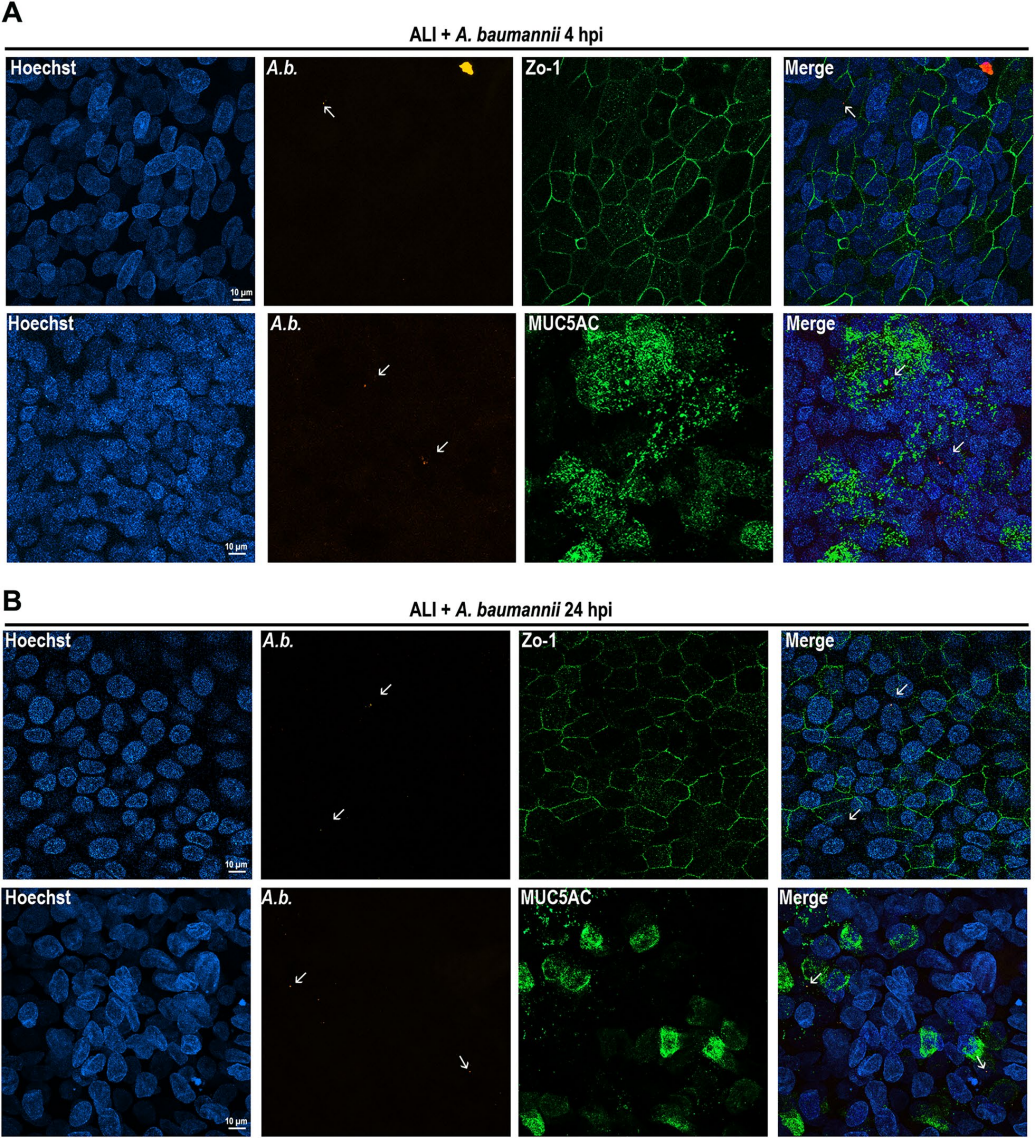
Immunofluorescence of ALI infected with *A. baumannii*. Representative images of infected ALI cultures stained with antibodies against *A. baumannii* (*A.b*., red) and either MUC5AC (green) or ZO-1 (green) at 4 and 24 h post-infection (hpi). Nuclei were counterstained with Hoechst 33342 (blue). Non-infected ALI cultures were treated with an equivalent volume of DMEM-F12 as a negative control. Three independent experiments were performed. Scale bar sizes are indicated in the images

Immunofluorescence (IF) staining was used to characterize specific interactions between the bacteria and epithelial cells. No clear co-localization was observed under our experimental conditions between bacteria and secreted mucin glycoproteins MUC5AC or the regulator of barrier function, zonulin (ZO-1). IF showed that *A. baumannii*-infected ALI exhibited a less defined pattern of ZO-1 immunostaining, particularly at 24 h post-infection when compared to unifected cells, indicating a less polarization state of the epithelial cells (Fig. 3 and Fig. S2). Interestingly, a reduced amount of excreted mucin was detected at 24 hpi, in accordance with their swollen and hypertrophic phenotype (Fig. 2B and D and Fig. 3B).

### *A. baumannii* infection induces airway epithelial barrier disruption

**Fig. 4:**
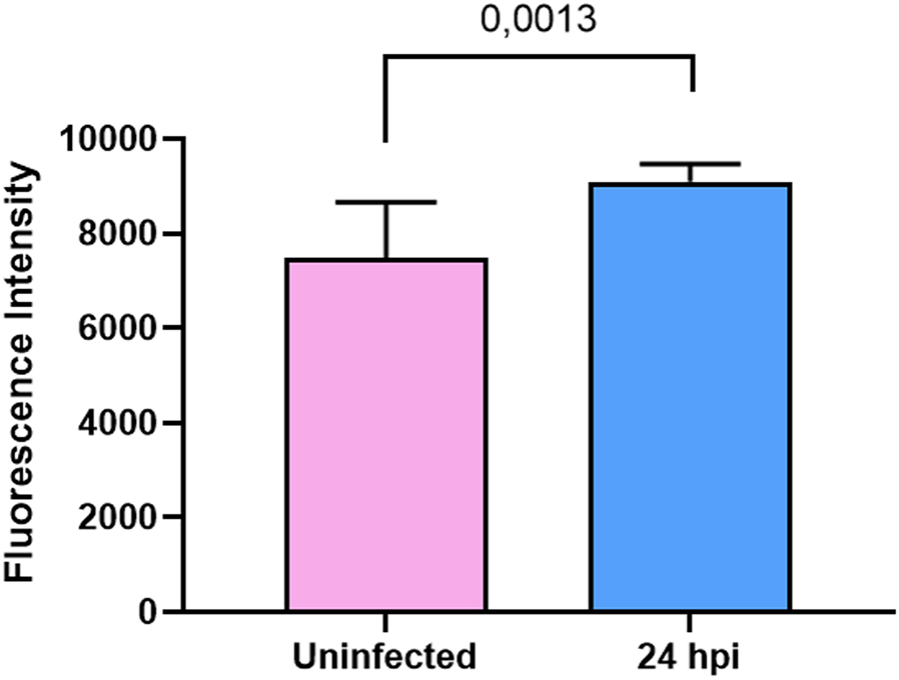
*A. baumannii* infection increases paracellular permeability in ALI cultures. Differentiated 2D ALI bronchial epithelial cultures were infected apically with strain AB507. At 24 h post-infection (hpi), barrier integrity was assessed by measuring the paracellular flux of 2 mg/ml of FITC-dextran (4 kDa) from the apical to the basolateral compartment. After 2 h of further incubation at 37 °C and 5% CO2, fluorescence measurement (excitation at 492 nm and emission at 520 nm) were recorded using a CLARIOStar microplate reader (BMG Labtech, Offenburg, Germany) in a 96-well black microtiter. The Adjust Gain setting was calibrated using a 2 mg/ml solution to standardize all sample readings. Data are expressed as mean ± standard deviation of three independent experiments. Statistical analysis was performed using Student’s t-test

To monitor the functional integrity of epithelial barrier, the paracellular FITC-dextran flux assays of infected and uninfected ALI cultures were performed at 4 and 24 hpi. Pairwise comparison indicated significant differences (*P* = 0.0013) between ALI cultures at 24 hpi (Fig. 4), indicating altered expression and/or redistribution of tight junction proteins and perturbations of the actin cytoskeleton and increased paracellular permeability in ALI infected cultures.

### *A. baumannii* infection triggers a high inflammation response in ALI cultures

**Fig. 5:**
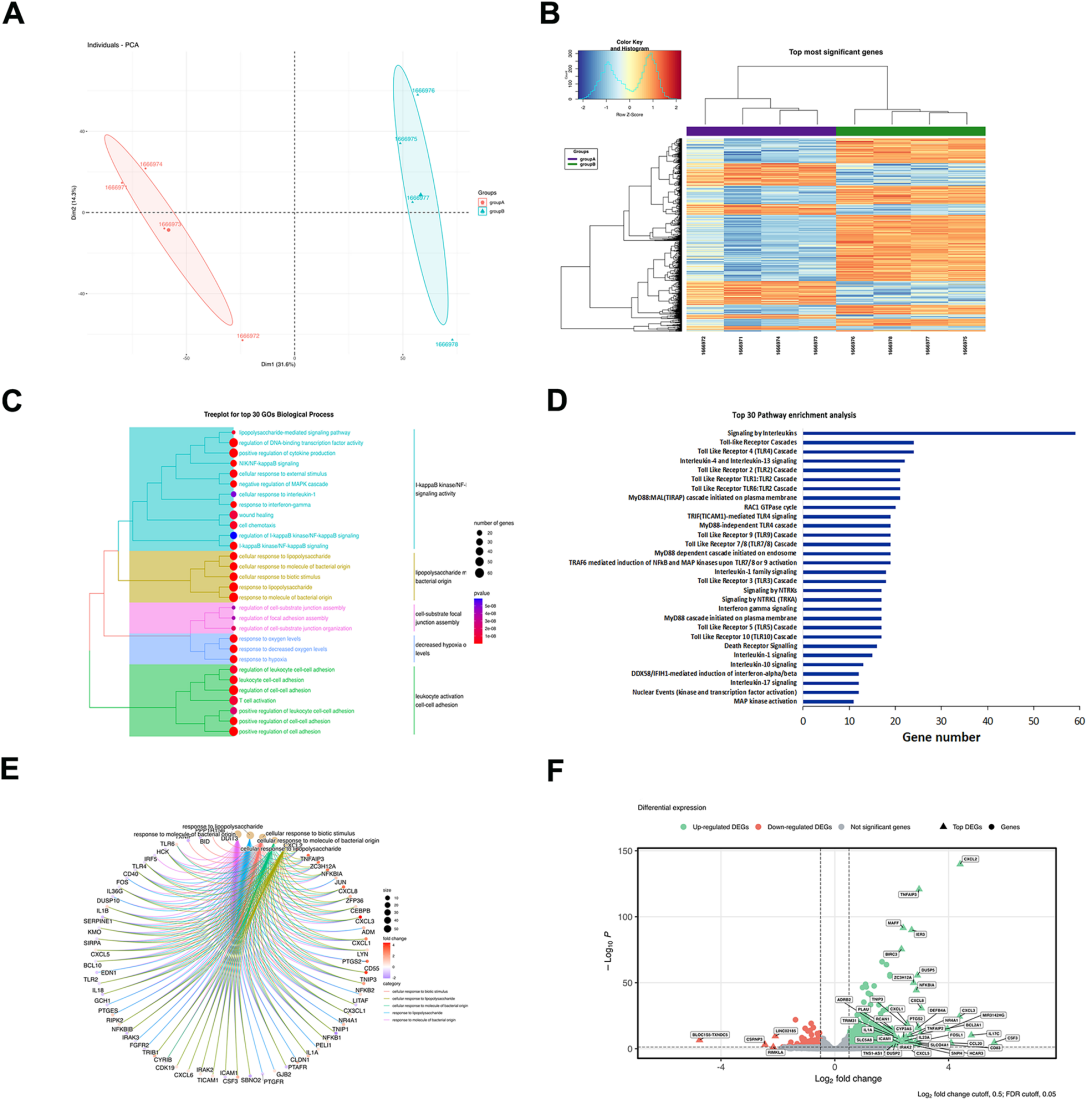
Global transcriptiomic response of bronchial epithelium to *A. baumannii* AB5075 infection at 4 hpi. **A** Principal Component Analysis (PCA) plot illustrating the clustering of unexposed (group A) and *A. baumannii*-exposed (group B) bronchial cells based on transcriptomic profiles. **B** Heatmap and hierarchical clustering dendrogram of the top differentially expressed genes (DEGs) in unexposed and exposed bronchial cells (FDR *P* value < 0.05). Warmer colors indicate upregulated genes, while cooler colors indicate downregulated genes. **C** Tree plot of the top 30 Gene Ontology (GO) terms related to the biological process of DEGs. The size of each node represents the level of enrichment significance, and the hierarchical arrangement reflects functional similarities between terms. **D** Description of the top 30 pathway enrichment analysis within the DEGs list, obtained through the use of a hypergeometric model. **E** Chord diagram illustrating the log_2_ fold changes of genes involved in the selected GO terms. **F** Volcano plot depicting the distribution of DEGs, with the most significantly upregulated (green) and downregulated (red) genes highlighted. The x-axis represents log2 fold change, and the y-axis represents -log10 (FDR *P* value), with a threshold of FDR < 0.05 used to identify significant DEGs

To investigate gene expression in ALI cultures following *A. baumannii* infection, we compared the transcriptional profiles of infected and non-infected bronchial cells using RNA-seq at 4 hpi, a time point when histological changes begin to emerge. The clear separation between the groups in the Principal-component analysis (PCA) plotting revealed high intra-group similarity and strong inter-group divergence (Fig. 5A). The analysis identified 1,099 statistically significant differentially expressed genes (DEGs) (*P* value ≤ 0.05), with 584 upregulated and 515 downregulated. The clustered heatmap of all DEGs is presented in Fig. 5B, and the complete DEG list is available in Table S2. When considering DEGs with a False Discovery Rate (FDR) ≤ 0.05, 441 genes were upregulated, and 227 were downregulated. The Gene Ontology (GO) enrichment analysis was conducted to elucidate the biological functions of DEGs upon bacterial exposure. The enriched GO terms in biological process included pathways related to inflammation and its regulation, immune response, cell–cell interaction, along with cell cycle progression, apoptosis, cellular stress, and metabolism (Fig. 5C and D). A strong immune response was evident, with differential expression of genes involved in interleukin-10 (IL-10), interleukin-4 (IL-4), interleukin-13 (IL-13), and interleukin-17 (IL-17) signaling, indicating a dynamic regulation of pro- and anti-inflammatory responses (*SOCS3, IL17C, IL1RN, IL4R*, *PTAFR*). Additionally, interleukin-1 (IL-1) processing and RIP-TRAF6-mediated NF-κB activation suggest a strong inflammatory response. Notably, the long non-coding RNA (lncRNA) MIR3142HG was also upregulated, potentially contributing to immune modulation in response to *A. baumannii* infection. Pathways involved in chemokine receptor signaling were also significantly enriched, highlighting the recruitment of immune cells to the site of infection (*NFKBIA, NFKBIB, NFKBIE, NFKBIZ, TNFAIP3, REL, BCL3*). TP53 downregulation, along with altered expression of death receptor-related genes, suggests apoptosis suppression or a shift toward survival mechanisms in response to bacterial infection. PI3K signaling dysregulation was observed, with downregulation of *PIK3R1, PIK3R2*, and *PTGS2*, and upregulation of *IRS1*/2, affecting cell survival and immune responses. Upregulation of *RND1, RND3, RHOB*, and *RHOBTB2* in the RAC1 GTPase cycle suggests cytoskeletal disruption, potentially weakening cell junctions and barrier integrity in bronchial epithelial cells. Additionally, *FGFR2* ligand binding (*FGFR2*), and ERK inactivation (*DUSP1, DUSP5, DUSP6*) were also detected (Fig. 5C and D). Furthermore, Fig. 5E illustrates the robust proinflammatory response to bacterial lipopolysaccharide and related molecules, with GO term connections representing shared genes. Notably, volcano plot analysis highlighted significantly altered genes in infected ALI cultures (Fig. 5F). Additionally, *IFITM2* and *IFNGR1*were induced, further amplifying the inflammatory cascade. Upregulation of *CSF3, ICAM1, MMP13, PLAU, PTGS2, VCL*, and *FERMT2* suggests active epithelial remodeling and barrier modulation. Conversely, downregulation of *PIK3R1, PIK3R2, GSTA2, TRIM25*, and *MAP3K1*, involved in oxidative stress defense and cytoskeletal regulation, may compromise epithelial resilience. To validate this result, we performed reverse transcriptase quantitative PCR (RT-qPCR) assays to assess the RNA transcript levels of genes representing crucial hubs in the three pathways (Table S1 and Fig. S3). Expression of those genes was in agreement with data from RNA-seq (R squared = 0,9771).

**Fig. 6:**
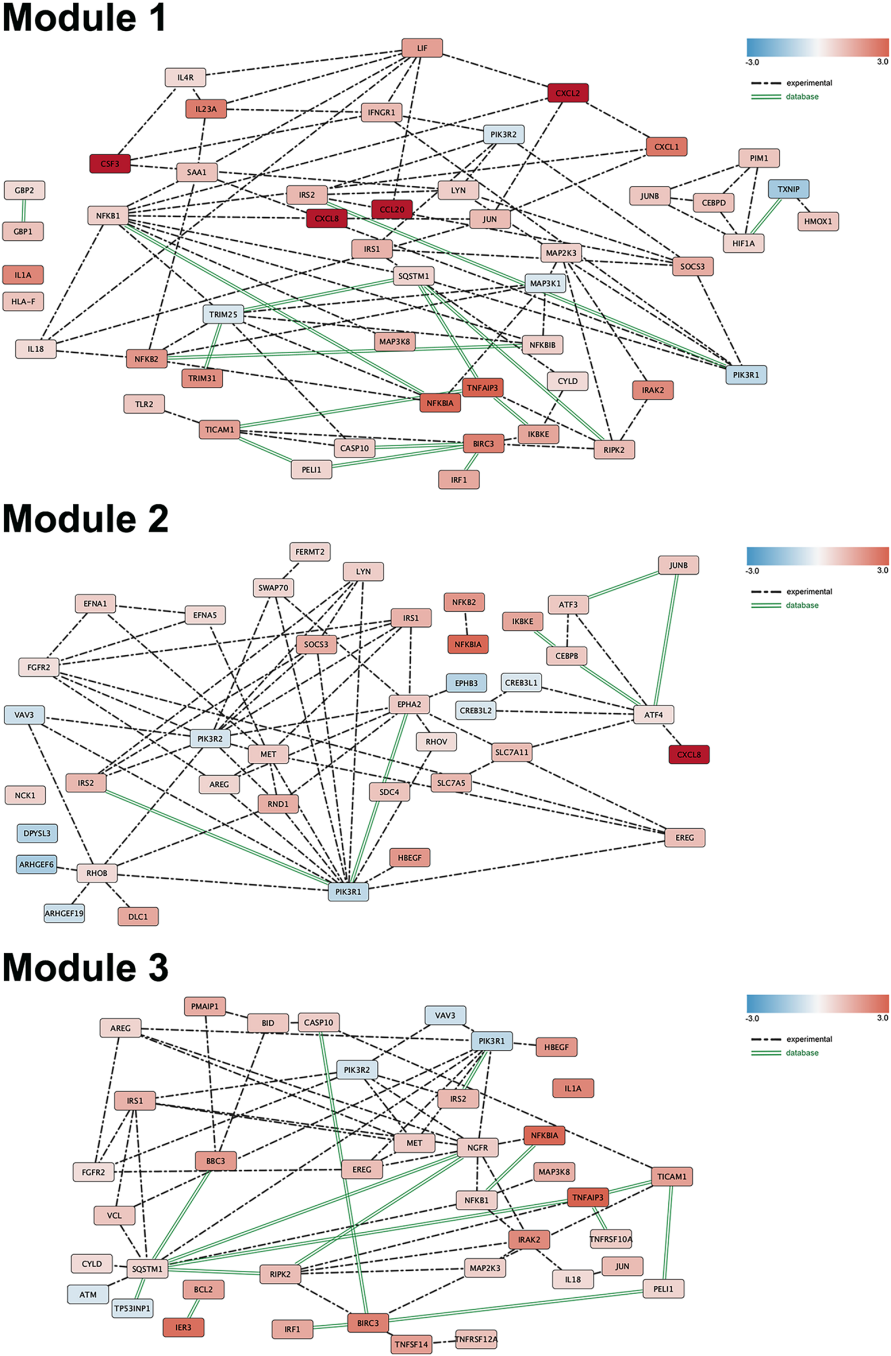
Protein–protein interaction network. The three modules represent the protein–protein interaction in the main pathways idendified constructed by Reactome functional interactions Cytoscape Plugin. Module 1, DEGs involved in inflammation and signaling regulation, module 2 DEGs involved in cell–cell interaction, module 3 DEGs in cell stress, survival and apoptosis. The color code indicates up o downregulation. Dotted lines reports experimentally demonstrated interactions, solid green lines interactions reported in the databases

To better understand the triggered pathways, the protein–protein interaction (PPI) network of DEGs was constructed by the STRING database and displayed via the Reactome FI Cytoscape Plugin. Three main modules were retrieved, each containing 35 to 39 nodes (Fig. 6). Interestingly, *PIK3R1* and *PIK3R2* emerged as key hubs in all modules; these phosphoinositide-3-kinases(PI3Ks) genes encode the regulatory subunits (p85α and p85β, respectively) of Class IA PI3Ks, which are ubiquitously expressed in the body and play crucial roles in signal transductions of cytoskeletal regulation, apoptosis, and immune responses [31]. The Insulin receptor substrate 2 (IRS2) was also present in all modules (Fig. 6). Module 1 highlights pro-inflammatory genes involved in cytokine signaling, and immune regulation, including *CXCL2, CXCL8, CCL20, IL18, IL23A,* and *LIF*, suggesting a robust pro-inflammatory response, through NF-κB (*NFKB1, NFKB2,* and *NFKBIB*) and JAK-STAT pathways. Moreover, Toll-Like Receptor (TLR) activation (*TLR2, TICAM1*, and *RIPK2*) suggests strong pattern recognition receptor (PRR) activation, leading to cytokine production and antimicrobial responses. Expression of *CSF3* (colony-stimulating factor 3) links immune activation, epithelial integrity, and cell survival during infection, playing a dual role in both host defense and tissue damage modulation. IRF1 and IFNGR1 support interferon signaling for antiviral and antibacterial defense. Expression of NF-κB regulators, *TNFAIP3* (A20) and *CYLD,* suggest a mechanism to prevent excessive inflammation and tissue damage, while *TXNIP* downregulation may reflect metabolic stress adaptation. The downregulation of *PI3K-*related genes (*MAP3K1, PIK3R1, PIK3R2, TRIM25*, and *TXNIP*) indicates PI3K-Akt inhibition, potentially shifting cellular priorities toward immune activation over cellular proliferation or survival. Module 2 focused on epithelial barrier function and growth factor signaling, identified *EREG, FGFR2,* and *MET* as central hubs, alongside *AREG* and *HBEGF*, which support epithelial repair and regeneration after infection-induced damage. Cytoskeletal remodeling and cell adhesion changes were indicated by upregulation of *RHOB, RND1, DLC1, RHOV, FERMT2, SDC4*, and downregulation of *ARHGEF6* and *ARHGEF19*. Interactions among *CXCL8, NFKB2, NFKBIA, SOCS3, ATF3, ATF4,* and *JUNB* suggest inflammation-induced epithelial damage. Module 3, related to cell survival and apoptosis, links *SQSTM1* (p62) to autophagy and inflammatory signaling. Apoptotic signals are supported by *BIRC3* and *CASP10*, while *TNFRSF10A* (TRAIL-R1) and *TNFRSF12A* indicate death receptor-mediated apoptosis. Expression of *BBC3* (PUMA), *BID*, and *PMAIP1* (NOXA) suggests activation of intrinsic and extrinsic apoptotic pathways. Downregulation of *ATM* and *TP53INP1*, critical for DNA damage repair, may indicate genomic instability and reduced cell viability. However, *BCL2* expression may counteract apoptosis, promoting cell survival. Growth factors *FGFR2, MET, AREG, EREG,* and *HBEGF* could support tissue repair, counterbalancing apoptosis with tissue repair and regeneration.

**Fig. 7:**
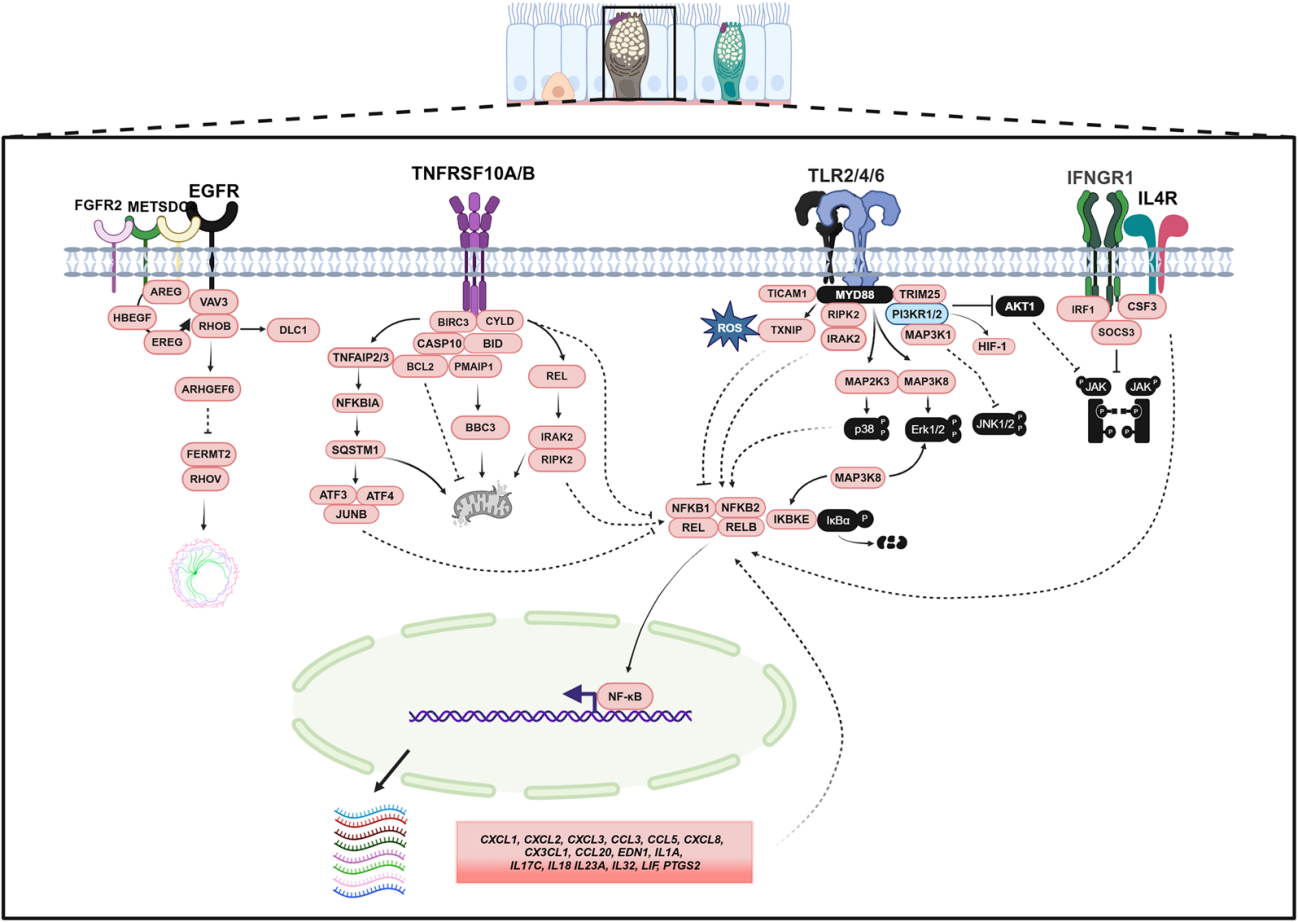
Proposed regulatory networks of human bronchial epithelium upon exposure to *A. baumannii*. Based on the results of this study, three main pathways highly interconneced were triggered by bacteria: inflammation and signaling regulation, cell–cell interaction, and autophagy/cell death. Black-filled proteins were not found among DEGs. Orange and light blue proteins represent upregulated and downregulated genes, respectively. Solid lines indicate previously demonstrated interactions, while dotted lines refer to missing interactors. Additional details are provided in the main text. The figure was created with BioRender.com

Integrating these data with the KEGG pathway and STRING database allowed us to generate a working model of the three main triggers elicited by *A. baumannii* during the exposure of bronchial epithelium (Fig. 7).

## Discussion

The airway epithelium serves as the first line of defense in the lungs, acting as a physical barrier against inhaled pathogens while also recruiting and activating effector cells to eliminate invaders. The bronchial epithelium is a pseudostratified structure primarily composed of columnar multiciliated, secretory (goblet), and undifferentiated cells that overlay smaller basal cells with self-renewal capacity, along with rarer cell types such as neuroendocrine and brush (tuft) cells [32]. In this study, we investigated the response of human bronchial epithelial cells to exposure to the opportunistic pathogen *A. baumannii*. To the best of our knowledge, this is the first study addressing this issue using a fully differentiated and functional human epithelium of the lower respiratory tract, serving as an ex vivo organotypic model (Fig. 1). As early as 4 hpi, mucus-secreting goblet cells appeared hypertrophic and secreted less mucin, the ZO-1 immunostaining pattern became less defined, and the epithelial barrier was compromised (Figs. 2 and 3). These changes progressively intensified in infected ALI cultures, becoming more pronounced at 24 hpi (Figs. 2 and 3), and were associated with increased permeability to FITC-dextran compared to uninfected ALI cultures (*P* = 0.0013) (Fig. 4). This recalls the goblet cell death and epithelial rupture reported for *P. aeruginosa* in an identical *in vitro* model, via the Type III Secretion System (T3SS) [20]; however, since *A. baumannii* lacks this virulence mechanism, it is likely that other secretion systems (*e.g*. the T6SS) or virulence factors are responsible for the epithelial damage observed in our study. In addition, the altered ZO-1 fluorescence pattern highlighted the different strategy used by *A. baumannii* compared to *Burkholderia cenocepacia*, which disrupts the cell-to-cell junctions at the occluding level, leaving ZO-1 unaffected [33]. Interestingly, we did not observe alterations in ciliated cells or the overexpression of Spondin 2 (SPON2), a pattern recognition receptor (PRR) molecule, known to be upregulated upon bronchial epithelial interaction with nontypeable *Haemophilus influenzae* [34]. Taken together, these findings indicate that *A. baumannii* adopts unique strategies to disrupt the airway epithelial integrity, underlining the importance of pathogen-specific studies and reliable *in vitro* models for accurately dissecting airway infection dynamics and host–pathogen interactions.

At the transcriptomic level, RNA sequencing and bioinformatics analyses identified 668 DEGs, with 441 upregulated and 227 downregulated in infected versus uninfected ALI cultures at 4 hpi. Three main pathways were detected (Fig. 6). Most upregulated genes were involved in the production and release of pro-inflammatory cytokines, chemokines, and immune mediators that facilitate immune cell recruitment, particularly neutrophils and macrophages, as previously reported [13]. This response is critical for pathogen clearance through phagocytosis and eventual bacterial killing. Several DEGs identified in this study overlapped with those reported in a previous study where human nasal epithelial cells were exposed to lipopolysaccharide (LPS) from *Escherichia coli* O55:B5 [35]. Common genes included *CSF3, CXCL1, CXCL8, CCL20, SAA1, BIRC3, IRAK2, NFKBIZ, ZC3H12A, RELB, IL1A*, and *JUN*, which are involved in chemokine-related pathways, such as the IL-17, TNF signaling pathways (Figs. 5, 6, and Table S2). Despite the structural differences between the lipooligosaccharide (LOS) of *A. baumannii* and the LPS of *E. coli* [35, 36], both bacteria appear to elicit a similar chemokine-driven immune response. In addition, in our study, TLR signaling plays a key role in pathogen recognition, detecting other bacterial-related structures, such as porin proteins, peptidoglycan, lipoproteins, and CpG DNA motifs (Figs. 6, and 7) [2, 4, 37]. The upregulation of the lncRNA MIR3142HG in response to *A. baumannii* LOS exposure further sustains the strong inflammation response observed, consistent with previous findings [38]. Studies in human pulmonary microvascular endothelial cells have shown that this lncRNA induces the release of inflammatory mediators, enhances apoptosis, and contributes to endothelial barrier disruption [38]. In agreement with the three main pathways identified in our study, the upregulation of MIR3142HG shows how deeply these host responses are interconnected. Additionally, among the upregulated genes directly and indirectly encoding macrophage and neutrophil chemoattractant proteins, we identified *CXCL1, CXCL2, CXCL3, CXCL5, CXCL6, CXCL8, CCL20, CX3CL1, CXCL16, CSF3, SAA1*, and *SAA2* (Table S2). These results are in agreement with those obtained from a mouse *in vivo* model, further supporting the strong host inflammatory response during *A. baumannii* infection [13]. Macrophages play a dual role in bacterial infections by directly defending against pathogens and promoting neutrophil recruitment [2, 4, 39]. Neutrophils are particularly critical in respiratory infections [2, 4, 40]. Upon infection, neutrophils are recruited to the site as early as 4 h, with infiltration peaking at 24 h post-infection. Notably, the strong upregulation of CXCL8 (IL-8) observed in this study is consistent with previous findings in lung cell lines [41, 42], further supporting its key role in recruiting neutrophils and monocytes during *A. baumannii* infection. While neutrophil influx is particularly needed for pathogen clearance, inflammation must be finely controlled to avoid tissue damage, and to allow effective repair and rebuilding of the tissue. Accordingly, our pathway enrichment analysis indicates that intact and viable *A. baumannii* cells trigger the expression of genes directly and indirectly involved in the production of type 2 cytokines, IL-4, and IL-13 (Fig. 5, and Table S2). In contrast to type 1 immune responses, type 2 immunity plays a dual role, being anti-inflammatory to prevent excessive tissue damage caused by inflammation, and tissue-reparative, to promote tissue repair [43]. Matrix metalloproteases (MMPs) play an essential role in the normal tissue repair process by degrading almost every component of the extracellular matrix components [43]. Research has shown that IL-13 and, to some extent, IL-4 can induce the expression and activity of MMP7 and MMP13 in airway epithelial cells [43–45]. These MMP-inducers were also found to facilitate recruitment of neutrophils to the sites of infection [43–45]. Interestingly, in an *in vivo* model of *A. baumannii* pneumonia, MMP9 and MMP14 were upregulated, whereas the Gram-negative bacterium *Francisella tularensis* induces the expression of MMP9 only *in vivo*; these proteins could be responsible for bacterial spread, severe lung damage due to neutrophil accumulation, and increased mortality [13, 46]. Furthermore, the opportunistic pathogen *P. aeruginosa* induces increased expression levels of MMP7 in human lung epithelial cells as early as 3 hpi, via flagellin signaling; the MMP7 upregulation may contribute to tissue damage and facilitate *P. aeruginosa* dissemination within injured tissues [47]. In addition, it has been reported that in *P. aeruginosa* infected differentiated human bronchial epithelial cell cultures, Th2 cytokines attenuates hBD-2 mRNA upregulation [43]. Conversely, in our experimental conditions, a net increase in defensin beta 4A (DEFB4A) mRNA was observed (Table S2), suggesting a different behavior of *A. baumannii* with respect to *P. aeruginosa*. The Th2 cytokine pathway could synergize with the upregulated and downregulated expression of 68 and 22 genes, respectively, that affect directly and indirectly epithelial barrier integrity, adhesion molecules, and cytoskeletal reorganization (Table S2). Genes like *ICAM1, SDC4, VCL, CLDN1, LAMB3, LAMC2, FERMT2, RND1, RND3, RHOV, RHOB, ARHGEF6, ARHGEF19, DLC1, DOCK4, PALLD, ITGB8, KRT6B, KRT17,* and *SEPTIN11* regulate cytoskeletal organization, cell junctions, and adhesion. Together with the downregulation of *KRT15, VPS13D,* and *SRGAP3*, these data suggest epithelial barrier dysfunction, reduced cytoskeletal stability, and increased permeability, facilitating bacterial invasion and dissemination. These results are in agreement with the goblet cells appearance as swollen, grainy, and irregular shape, and corroborate the severe impact of *A. baumannii* on the overall architecture of ALI cultures (Figs. 2, 6 and 7). Moreover, the upregulation of *MMP7, MMP13* as well as *MET, EPHA2, EFNA1, EFNA5, HBEGF, FGFBP1, TNS1, TNS4, PLAUR,* and *PTGES* as well as the downregulation of *PIK3R2, MAP3K1, CREB3L1, CREB3L2,* and *IRX5* could participate to extracellular matrix degradation. Noteworthy, these latter genes could also impair pro-survival and regenerative pathways [48]. Interestingly, also *PI3KR1* and *PIK3R2* were downregulated in our experimental conditions (Figs. 6, 7 and Table S2), in agreement with previous finding on *A. baumannii* infection *in vivo* [13]. Both genes encode regulatory subunits of Class IA phosphoinositide 3-kinases (PI3Ks), that mediates the synthesis of phosphatidylinositol (3,4,5)-trisphosphate (PIP_3_) within cells. This lipid second messenger activates the PI3K-Akt pathway, a major survival signaling cascade. Among other effects, inhibition of this pathway can induce apoptosis [48, 49]. This pathway is subverted by several human pathogens for their advantage. For instance, *Salmonella* and *Listeria monocytogenes* hijack the PI3K-Akt pathway to prevent host cell apoptosis, creating a protected intracellular niche for their survival [50–52]. In a human lung tissue explant model, Sohail et al. suggested that activation of the PI3K-Akt pathway could be modulated by *P. aeruginosa*-derived microRNAs targeting host mRNAs, thereby contributing to a rapid pro-inflammatory response, early epithelial barrier disruption, and activation of tissue damage-associated pathways [53]. Conversely, enteropathogenic *E. coli* [54], *Streptococcus pneumoniae* [55], *Klebsiella pneumoniae* [56], and *Bacillus anthracis* [57] inhibit the PI3K-Akt pathway to enhance bacterial survival and dissemination by promoting autophagy/cell death and disruption of epithelial barriers. This finding is consistent with previous studies demonstrating that *A. baumannii* infection induced apoptosis, pyroptosis, and necroptosis across different cell models, regardless of the strain used [58–62]. However, unlike a previous *in vivo* study [13], we did not observe JAK-STAT pathway activation. This discrepancy may be due to the earlier time point of our RNA-seq analysis (4 hpi vs. 24 hpi in the previous study), as JAK-STAT activation could be a later event in the host response. A limitation of our model is the lack of immune cells that are key producers of JAK-STAT-activating cytokines, which may further contribute to the absence of its activation. Finally, it is important to note differences from our previous studies using carcinogenic human lung epithelial cells, emphasizing the distinct host response observed in a more physiologically relevant model [42, 63]. At the same time, our findings reinforce the reliability of ALI cultures in dissecting the complex interplay between *A. baumannii* and the host. Despite some limitations (a single donor and one bacterial strain), a major strength of this study is the early analysis of the host response, which closely aligns with *in vivo* studies and provides crucial insights into the initial stages of infection.

## Conclusion

This study highlights the early host response to *A. baumannii* using a physiologically relevant air–liquid interface (ALI) bronchial epithelium model. Within 4 hpi, *A. baumannii* triggered a strong inflammation, induced apoptosis/cell death, compromised cytoskeletal organization, leading to increased permeability and early tissue damage. By 24 hpi, goblet cell hypertrophy, reduced mucin secretion, and compromised epithelial integrity were highly evident. RNA sequencing revealed 668 DEGs across inflammation, epithelial barrier disruption, and apoptosis/cell death pathways, with a strong expression of pro-inflammatory chemokines (IL-8/CCL20) and type 2 cytokines (IL-4, IL-13), indicating that *A. baumannii* infection triggers both innate immune recruitment and broader inflammatory signaling. Significant alterations in cell adhesion and extracellular matrix genes suggest bacterial dissemination, while PI3K-Akt pathway inhibition aligns with other pathogens promoting cell death and barrier dysfunction. This work establishes a strong foundation for further investigations into *A. baumannii*-host interactions using a robust and reproducible human *in vitro* model. However, future studies including innate immune cells and focusing on cellular pathway herein described will be essential to fully elucidate the mechanisms underlying *A. baumannii* pathogenesis.

## Supplementary Information

Supplementary Material 1: Fig. S1. Histology of ALI infected with *A. baumannii*. A total of 20 µl of bacteria resuspended in DMEM-F12 was used for infection at an MOI of 100. Non-infected ALI cultures were treated with an equivalent volume of DMEM-F12 as a negative control. (A, B) Representative images of infected and uninfected ALI cultures stained with H&E at 4 hours post-infection (hpi) and (C, D) at 24 hpi, respectively.

Supplementary Material 2: Fig. S2. Immunofluorescence of uninfected ALI cultures. ALI cultures were treated with 20 µl of DMEM-F12, and stained with antibodies against *A. baumannii* (*A.b*.) and either MUC5AC (green) or ZO-1 (green) at 24 h. Nuclei were counterstained with Hoechst 33342 (blue). Three independent experiments were performed. Scale bar sizes are indicated in the images.

Supplementary Material 3: Fig. S3. Comparison of RNA-seq and RT-qPCR data. RT-PCR analysis of *PIK3R1, PIK3R2, NFKBIA, CXCL8, MAP3K1, SWAP70, RHOB, CASP10, IRAK2*, and *SOC3* in infected and uninfected ALI cultures of human bronchial epithelial cells. GAPDH was used as a reference gene. Each group contained three biological sample repeats, and each sample contained four technical repeats of qRT-PCR. A Pearson correlation coefficient of 0.9771 was observed between the RNA-seq and RT-qPCR data expressed as log_2_ fold change.

Supplementary Material 4.

Supplementary Material 5.

Supplementary Material 6.

## Data Availability

The RNA-seq data of this study have been deposited in NCBI underBioProject No. PRJNA1219108.

## Abbreviations

ALI: Air–liquid interface
CFU: Colony forming units
DEGs: Differentially expressed genes
FDR: False rate discovery
FITC: Fluorescein isothiocyanate
H&E: Hematoxylin–eosin
hpi: Hours post-infection
IIF: Indirect immunofluorescence
IL: Interleukin
LB: Luria–Bertani
LOS: Lipooligosaccharide
LPS: Lipopolysaccharide
MOI: Multiplicity of infection
MUC5AC: Mucin-5 subtype AC
NHBE: Normal human bronchial epithelial cells
PAS: Periodic acid–Schiff
PBS: Phosphate-buffered saline
RT: Room temperature
RT-PCR: Reverse transcriptase-polymerase chain reaction
ZO-1: Zonulin
