# Supplementary figures and images for: Goblet cell breakdown: transcriptomics reveals Acinetobacter baumannii early and robust inflammatory response in differentiated human bronchial epithelial cells
Source: J Biomed Sci. 2025 Jul 9;32:63. doi: 10.1186/s12929-025-01159-1 (PMC12239265; doi:10.1186/s12929-025-01159-1)

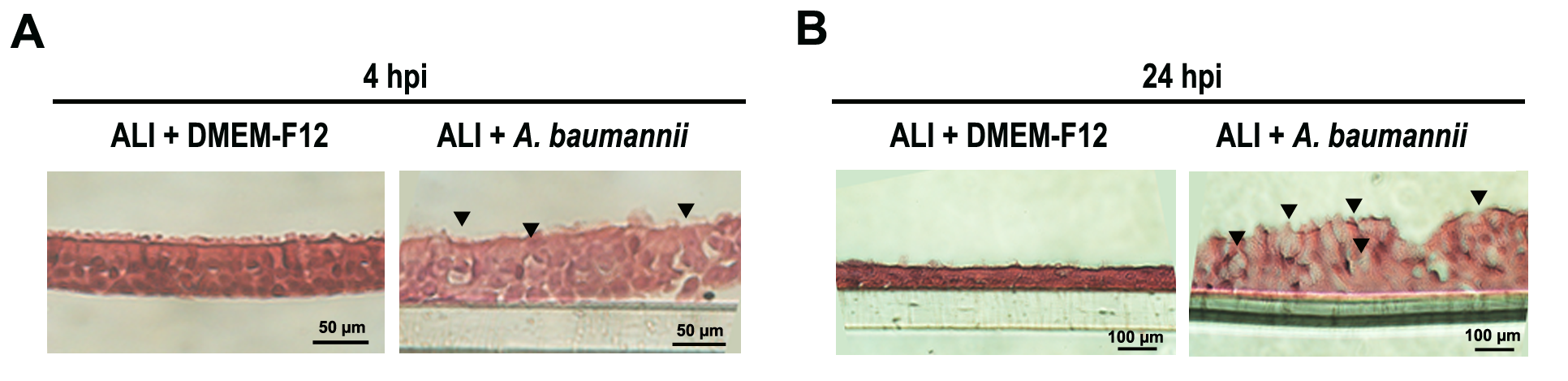

Supplement: Supplementary file 1 — Supplementary Material 1: Fig. S1. Histology of ALI infected with A. baumannii. A total of 20 µl of bacteria resuspended in DMEM-F12 was used for infection at an MOI of 100. Non-infected ALI cultures were treated with an equivalent volume of DMEM-F12 as a negative control. (A, B) Representative images of infected and uninfected ALI cultures stained with H&E at 4 hours post-infection (hpi) and (C, D) at 24 hpi, respectively. [file 12929_2025_1159_MOESM1_ESM.tif]

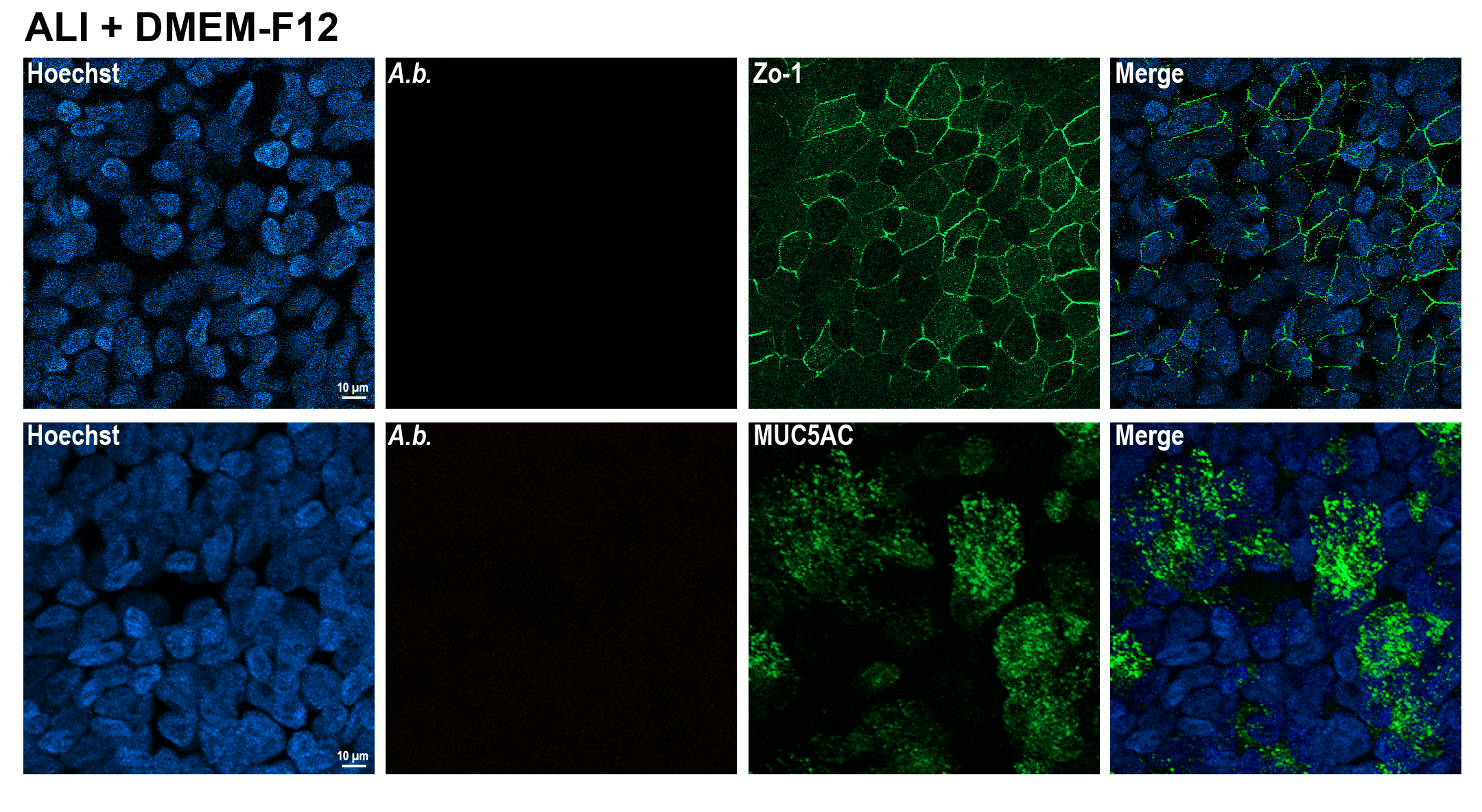

Supplement: Supplementary file 2 — Supplementary Material 2: Fig. S2. Immunofluorescence of uninfected ALI cultures. ALI cultures were treated with 20 µl of DMEM-F12, and stained with antibodies against A. baumannii (A.b.) and either MUC5AC (green) or ZO-1 (green) at 24 h. Nuclei were counterstained with Hoechst 33342 (blue). Three independent experiments were performed. Scale bar sizes are indicated in the images. [file 12929_2025_1159_MOESM2_ESM.tif]

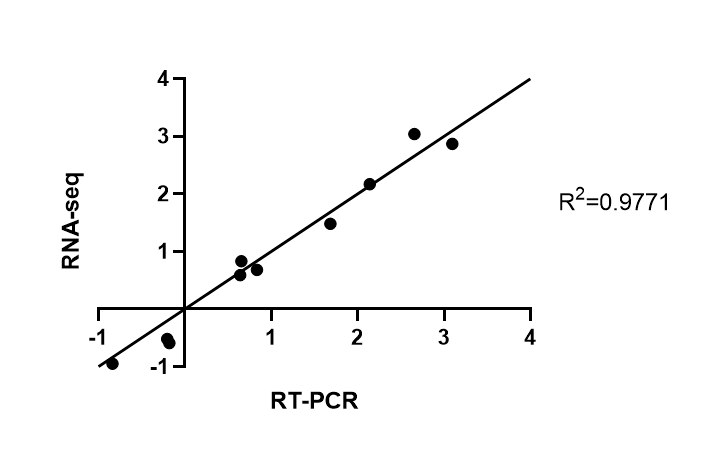

Supplement: Supplementary file 3 — Supplementary Material 3: Fig. S3. Comparison of RNA-seq and RT-qPCR data. RT-PCR analysis of PIK3R1, PIK3R2, NFKBIA, CXCL8, MAP3K1, SWAP70, RHOB, CASP10, IRAK2, and SOC3 in infected and uninfected ALI cultures of human bronchial epithelial cells. GAPDH was used as a reference gene. Each group contained three biological sample repeats, and each sample contained four technical repeats of qRT-PCR. A Pearson correlation coefficient of 0.9771 was observed between the RNA-seq and RT-qPCR data expressed as log2 fold change. [file 12929_2025_1159_MOESM3_ESM.tif]
