# Supplementary material for: Goblet cell breakdown: transcriptomics reveals Acinetobacter baumannii early and robust inflammatory response in differentiated human bronchial epithelial cells
Source: J Biomed Sci. 2025 Jul 9;32:63. doi: 10.1186/s12929-025-01159-1 (PMC12239265; doi:10.1186/s12929-025-01159-1)
Supplement: Supplementary file 4 — Supplementary Material 4. [file 12929_2025_1159_MOESM4_ESM.docx]

Table S1. Primers used to validate RNA-seq data by qRT‐PCR analysis.

| ID* | SYMBOL | Module 1 | Module 2 | Module 3 | Primer 5’🡪3’ |
| --- | --- | --- | --- | --- | --- |
| ENSG00000145675 | PIK3R1 | 🗷 | 🗷 | 🗷 | FW CTCTCTGAAAGAACTGGTGCTACATT  RV AACGACTCCCTCAATGTCACACTA |
| ENSG00000105647 | PIK3R2 | 🗷 | 🗷 | 🗷 | FW CCCGGCAGAAGAAAATCAAC  RV TCTTGCCCACGTACCAAGTG |
| ENSG00000100906 | NFKBIA | 🗷 | 🗷 | 🗷 | FW GCTGAAGAAGGAGCGGCTACT  RV TCGTACTCCTCGTCTTTCATGGA |
| ENSG00000169429 | CXCL8 | 🗷 |  |  | FW ACCGGAAGGAACCATCTCACT  RV ATCAGGAAGGCTGCCAAGAG |
| ENSG00000095015 | MAP3K1 | 🗷 |  |  | FW AATCACACCACCCCGAAGAG  RV AACACGGCGGTTTGTTTCC |
| ENSG00000133789 | SWAP70 | 🗷 | 🗷 | 🗷 | FW CAGCCCTCCACCACACAAA  RV TGTCGCTCCAGTTCCTCTTGTT |
| ENSG00000143878 | RHOB |  | 🗷 |  | FW GACTCCCGCCCAAGCAT  RV CCCCAAGTCAGTTGCAAATGT |
| ENSG00000003400 | CASP10 |  |  | 🗷 | FW CTGGCAGAACTCCTCTATATCATACG  RV CGTAGAGCAGGTTTCTAAACAGAGAA |
| ENSG00000134070 | IRAK2 | 🗷 |  | 🗷 | FW CCCCAGCAGATTCCATTACCT  RV TGCTATGGCATTGCAGAACTG |
| ENSG00000184557 | SOC3 |  | 🗷 |  | FW TGGGACGATAGCAACCACAA  RV CGAAGTGTCCCCTGTTTGGA |

*Up and downregulated genes are reported in green and red, respectively.
